# Warming rewires nitrogen dynamics in rice

**DOI:** 10.1093/plphys/kiag390

**Published:** 2026-06-17

**Authors:** Neeta Lohani, Palak Chaturvedi

**Affiliations:** Assistant Features Editor, Plant Physiology, American Society of Plant Biologists; Department of Biotechnology, Thapar Institute of Engineering and Technology, Bhason Rd, Prem Nagar, Patiala, Punjab 147004, India; Molecular Systems Biology Lab (MOSYS), Department of Functional and Evolutionary Ecology, University of Vienna, Vienna Austria

Every grain/seed owes much of its protein to a dying leaf. As leaves age, they break down their chloroplasts, which store over 70% of leaf nitrogen, and export the released amino acids to developing grains (Avila-Ospina et al. 2014). In rice, this process alone supplies roughly 60% of all grain nitrogen (Masclaux-Daubresse et al. 2010). It is orderly, essential, and under normal conditions, gradual. However, warming alters this dynamic. Elevated temperatures during grain filling can push the leaves into early senescence, accelerating the export of nitrogen. The result is paradoxical: while yields may decrease, the grain protein content increases (Impa et al. 2021). With global temperatures projected to increase by 1.0 to 5.7 °C by the end of this century, this trade-off will have direct implications on the nutritional quality of rice. Not all grains in a rice panicle, however, respond identically to this heat-induced change. Superior spikelets (SS) on the upper primary branches flower first and fill fast. Inferior spikelets (IS) on the lower secondary branches flower later and fill poorly (Zhang et al. 2012). Do both types of grain benefit equally from the accelerated nitrogen delivery caused by heat?

In a recent article published in *Plant Physiology*, Zhao and colleagues (2026) investigated how temperature increases affect rice grain filling through a field warming experiment combined with nitrogen fertilization. They grew the heat-sensitive japonica cultivar Wuyujing 3 under a Free Air Temperature Enhancement (FATE) facility that raised canopy temperatures by 2.28 °C during the day and 4.33 °C at night throughout grain filling (from flowering until 60 days after flowering [DAF]). These temperature increases fall within the projected range by the Intergovernmental Panel on Climate Change (IPCC) and reflect the well-documented trend of greater warming at night than during the day due to climate change. Alongside warming, the authors applied an additional 60 kg N ha^−1^ at the onset of the temperature treatment to test whether nitrogen could influence the warming response. They monitored leaf physiology, nitrogen metabolism, and grain composition in both SS and IS across 10 time points from 3 to 45 DAF. Furthermore, they conducted untargeted metabolomic profiling of leaves, SS, and IS at 15 and 30 DAF.

Elevated temperatures had contrasting effects on the 2 types of spikelets. In SS, grain weight was significantly decreased by 2.1% and, while the final protein content at maturity was unchanged, warming significantly suppressed protein accumulation at 25 and 30 DAF during grain filling. In IS, both grain weight and final protein content increased significantly, by 4.23% and 2.95%, respectively, with the significant protein increase becoming apparent from 30 DAF onward. Exposure to high temperature also reduced the seed-setting rate by 16.08% and overall yield by 18.98%. Additional nitrogen raised protein content in both SS and IS regardless of temperature, with the highest accumulation under the combined warming and nitrogen treatment. However, it is important to note that this increase in protein came at a cost: the extra nitrogen further reduced grain weight in both spikelet types under elevated temperatures.

To investigate the source of the additional protein in IS, the authors conducted a detailed examination of the flag leaf. Elevated temperatures triggered oxidative stress starting at 9 DAF. The levels of hydrogen peroxide (H_2_O_2_) increased by 28% at 9 DAF and by 61% at 15 DAF, while the maximum quantum efficiency of photosystem II (Fv/Fm), a reliable indicator of stress, showed a significant decline from 9 to 30 DAF. During this period, the leaf also enhanced its nitrogen remobilization process. The activities of glutamine synthetase (GS) and glutamate synthase (GOGAT), enzymes responsible for converting ammonium into transportable amino acids such as glutamine and asparagine, increased significantly at select time points during early grain filling, with GS activity rising by 29% to 34% at 9 and 15 DAF (but not at 12 DAF), and GOGAT activity surging significantly by 91% to 128% at 12 and 15 DAF. The expression of amino acid transporters OsLHT1 and OsAAP6, which facilitate the movement of amino acids from leaves to grains (Peng et al. 2014; Guo et al. 2020), were upregulated during early grain filling: OsLHT1 significantly at 9 DAF, and OsAAP6 from 9 to 20 DAF. Free amino acids accumulated transiently in the leaf at 12 and 15 DAF and were also elevated in the stem sheath during this period, indicating active nitrogen export from source to sink. Net photosynthesis in the flag leaf began to decline from 12 DAF, and leaf chlorophyll and nitrogen content decreased from 20 DAF onward, marking the visible onset of senescence. The key insight from these observations is the timing. Warming prompted rapid nitrogen export from the flag leaf between 9 and 15 DAF, well before any visible signs of senescence appeared at 20 DAF.

Untargeted metabolomics revealed why SS and IS responded differently to this shared nitrogen supply. In SS, elevated temperature suppressed 3 key intermediates of the pentose phosphate pathway at both 15 and 30 DAF: β-D-fructose 6-phosphate, D-erythrose 4-phosphate, and D-glucose 6-phosphate. This pathway provides NADPH for reactive oxygen species (ROS) scavenging and supplies precursors for aromatic amino acid biosynthesis (Sharkey 2021). Its suppression in SS compromises both heat tolerance and the raw materials for protein synthesis. Indole-3-acetic acid (IAA) also dropped in SS at 15 DAF, which may weaken the apical dominance that SS normally exert over IS during grain filling. In contrast, IS demonstrated an opposite metabolic response. L-histidine, an amino acid involved in both protein synthesis and stress tolerance, rose by 65% at 15 DAF. D-raffinose, an osmoprotectant linked to heat, cold, and drought tolerance (Liu et al. 2023), accumulated to 1.7-fold at 15 DAF and 2.1-fold at 30 DAF compared to the control. Gentisic acid, a salicylic acid derivative that activates plant defense responses, also increased in IS at 15 DAF. Together, these metabolic changes suggest that the IS mount a coordinated stress protection program, allowing them to utilize incoming amino acids for protein synthesis rather than simply surviving heat damage.

The addition of nitrogen created its own distinct but related effects. It delayed leaf senescence, maintained higher leaf chlorophyll and nitrogen content, and raised grain protein in both SS and IS. However, it also led to a further reduction in grain weight under warming conditions. The authors suggest that delayed senescence slows the timely release of amino acids to grains and worsens the carbon to nitrogen imbalance that warming creates, particularly when carbon metabolism is already impaired by heat. This situation underscores a practical challenge for rice management: while nitrogen supplementation can enhance grain protein in warmer conditions, it does so at the cost of grain weight and overall yield.

The study by Zhao et al. (2026) presents 3 significant contributions (Fig. 1). First, it demonstrates that warming initiates an active and organized nitrogen remobilization program in the flag leaf before visible senescence occurs, rather than merely speeding up passive decay. Second, it reveals that the superior (SS) and inferior (IS) spikelets within the same panicle exhibit fundamentally different metabolic responses to heat. Specifically, IS spikelets implement a protective program that involves L-histidine, raffinose, and gentisic acid, which supports continued protein accumulation. Third, the study identifies *OsLHT1* and *OsAAP6* as key components in the leaf-to-grain nitrogen delivery system under warming conditions. Several questions arise from these findings. How much of the advantage seen in IS spikelets is due to their lower position in the canopy and likely cooler grain temperatures. Can post-anthesis root nitrogen uptake be adjusted to promote grain protein without compromising yield? Additionally, can the stress-protective metabolites identified in IS serve as selection markers for breeding climate-resilient cultivars?

**Figure 1:**
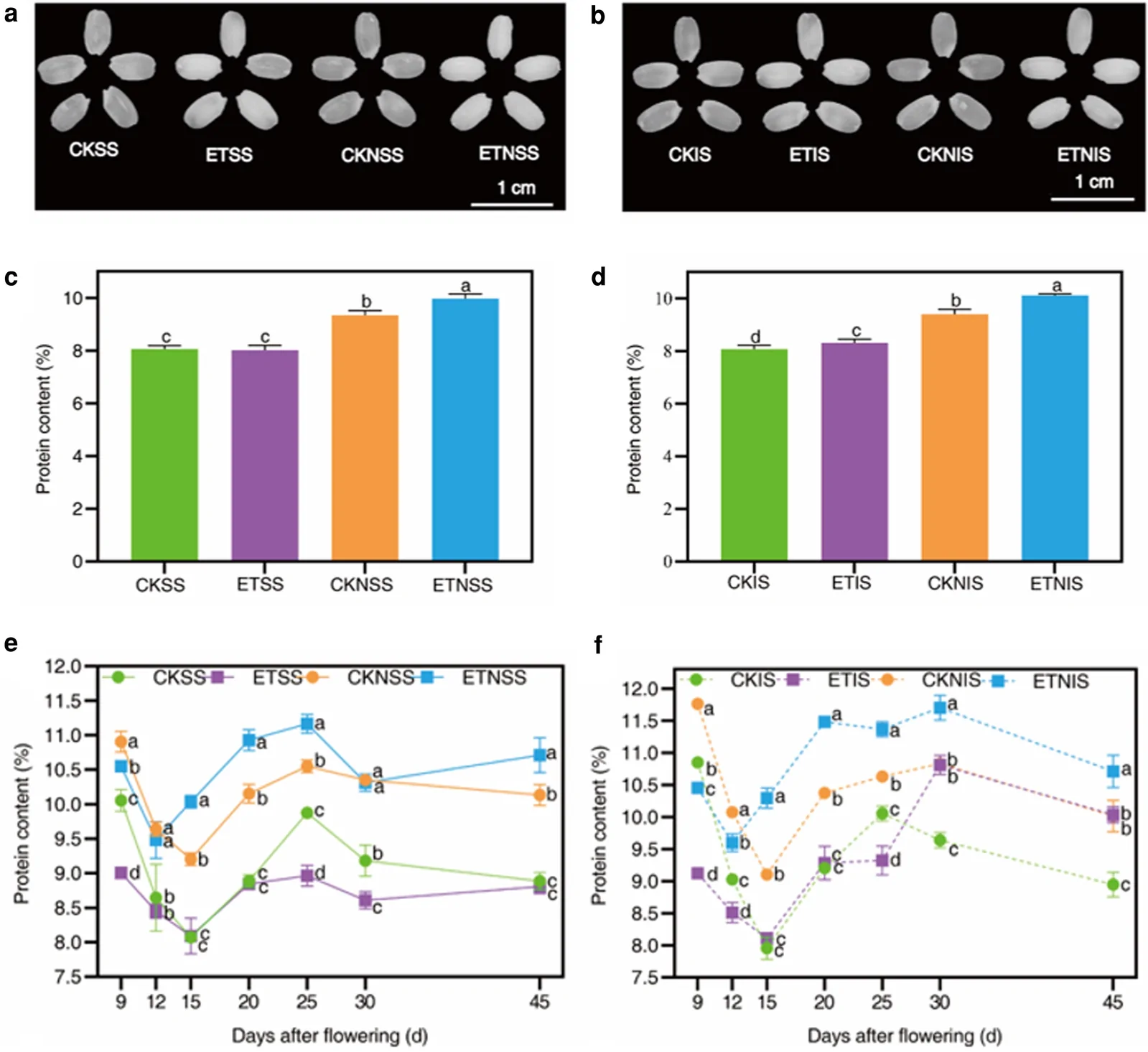
Warming reshapes grain protein accumulation differently in superior and inferior spikelets of rice. (a) Scanning images of mature brown rice for superior spikelets (SS) under natural temperature (CKSS), elevated temperature (ETSS), additional nitrogen under natural temperature (CKNSS), and additional nitrogen under elevated temperature (ETNSS). (b) Scanning images of mature brown rice for inferior spikelets (IS) under the corresponding treatments (CKIS, ETIS, CKNIS, ETNIS). (c) Grain protein content of SS at maturity. (d) Grain protein content of IS at maturity. (e) Dynamics of grain protein content during grain filling (9–45 DAF) in SS. (f) Dynamics of grain protein content during grain filling (9–45 DAF) in IS. Adapted from Zhao et al. (2026).

Future research utilizing isotope labeling and metabolic flux analysis, as the authors suggest, will be necessary to precisely map the nitrogen transfer from leaves to individual grain populations. For now, this study makes a compelling case that an accelerated nitrogen delivery schedule does not benefit all grains equally, and surprisingly, inferior spikelets may become the unexpected beneficiaries of warming.

**Recent related articles published in *Plant Physiology***

- Sandhu et al. (2024) identified natural variation at the *LONELY GUY-Like 1* (*LOGL1*) locus that regulates rice single grain weight under high night temperature stress through the thiamin biosynthesis pathway, providing a genetic basis for the warming-induced grain weight penalty observed in superior spikelets in the present study.
- Habiba et al. (2025) showed that the rice transcription factor *OsS40-14* coordinates chloroplast and reactive oxygen species gene networks to promote dark-induced leaf senescence in flag leaves, complementing the present finding that heat-induced ROS accumulation and chlorophyll loss drive nitrogen remobilization from leaves to developing grains.

## Data Availability

No new data were generated or analyzed in support of this article.
